# Childhood Diarrhea Exhibits Spatiotemporal Variation in Northwest Ethiopia: A SaTScan Spatial Statistical Analysis

**DOI:** 10.1371/journal.pone.0144690

**Published:** 2015-12-21

**Authors:** Muluken Azage, Abera Kumie, Alemayehu Worku, Amvrossios C. Bagtzoglou

**Affiliations:** 1 Ethiopian Institute of Water Resources, Addis Ababa University, Addis Ababa, Ethiopia; 2 School of Public Health, College of Health Sciences, Addis Ababa University, Addis Ababa, Ethiopia; 3 Department of Civil and Environmental Engineering, School of Engineering, University of Connecticut, Storrs, CT 06269, United States of America; University of South Africa (South Africa) and University of Tennessee (USA), UNITED STATES

## Abstract

**Background:**

Childhood diarrhea continues to be a public health problem in developing countries, including Ethiopia. Detecting clusters and trends of childhood diarrhea is important to designing effective interventions. Therefore, this study aimed to investigate spatiotemporal clustering and seasonal variability of childhood diarrhea in northwest Ethiopia.

**Methods:**

Retrospective record review of childhood diarrhea was conducted using quarterly reported data to the district health office for the seven years period beginning July 1, 2007. Thirty three districts were included and geo-coded in this study. Spatial, temporal and space-time scan spatial statistics were employed to identify clusters of childhood diarrhea. Smoothing using a moving average was applied to visualize the trends and seasonal pattern of childhood diarrhea. Statistical analyses were performed using Excel^®^ and SaTScan programs. The maps were plotted using ArcGIS 10.0.

**Results:**

Childhood diarrhea in northwest Ethiopia exhibits statistical evidence of spatial, temporal, and spatiotemporal clustering, with seasonal patterns and decreasing temporal trends observed in the study area. A most likely purely spatial cluster was found in the East Gojjam administrative zone of Gozamin district (LLR = 7123.89, p <0.001). The most likely spatiotemporal cluster was detected in all districts of East Gojjam zone and a few districts of the West Gojjam zone (LLR = 24929.90, p<0.001), appearing from July 1, 2009 to June 30, 2011. One high risk period from July 1, 2008 to June 30, 2010 (LLR = 9655.86, p = 0.001) was observed in all districts. Peak childhood diarrhea cases showed a seasonal trend, occurring more frequently from January to March and April to June.

**Conclusion:**

Childhood diarrhea did not occur at random. It has spatiotemporal variation and seasonal patterns with a decreasing temporal trend. Accounting for the spatiotemporal variation identified in the study areas is advised for the prevention and control of diarrhea.

## Introduction

The burden of communicable disease continues to be a public health problem in developing countries. The distribution of disease in the population is known to differ among groups, with children being the most vulnerable. Diarrhea, a gastrointestinal infection, is caused by a wide range of pathogens, including bacteria, viruses and protozoa [1, 2], and is often transmitted to humans via food or water that has been contaminated with fecal matter [2]. The disease is a major public health burden throughout the world, particularly in developing countries [3, 4], and children are severely and disproportionately affected. A 2012 review article on low and middle income countries indicated that childhood diarrhea had been reduced only modestly from nearly 1.9 billion episodes in 1990 to nearly 1.7 billion episodes in 2010 [5]. Diarrhea is also a major public health burden in Sub-Saharan Africa (SSA) countries [6], and was responsible for more than 50% of childhood morbidity and 50–80% of childhood mortality in the region [3, 4].

As a Sub-Saharan African country [7], Ethiopia has experienced childhood diarrhea and the associated high morbidity and mortality [4, 8]. Although studies conducted on childhood diarrhea morbidity have shown a slight decline in the past decade, the national prevalence of diarrhea has remained high in comparison to other developed countries. In the two weeks preceding the Ethiopian Demographic Health Survey conducted in 2000, childhood diarrhea prevalence was 24%; in 2005, it was 18%; and in 2011, it was 13.5% [9–11]. Moreover, other community-based studies conducted in different parts of the country at various times have shown the prevalence of diarrheal disease in the range of 11–35% [12–16]. Facility-based reports indicate it has been responsible for 11–18% of hospital admissions from all childhood diseases in the period between 2010 to 2012 [17, 18], and a community-based study in southern Ethiopia revealed 30% of child mortality was caused by diarrhea [19].

Childhood diarrhea incidence varies among countries as well as among the different regions of a given country. These differences can be attributed to climatic, environmental, behavioral, socio-demographic, and economic factors [20–22]. In particular, the disease has been widespread in areas where water resources are scarce and unsafe drinking water supplies, poor hygiene, and lack of sanitation are prevalent [23].

These factors have also favored the occurrence of clusters of childhood diarrhea cases in space and time. The understanding of the epidemiology and the disease pattern in time and space [24] is needed to manage diarrheal diseases. Thus, the identification of geographical areas with ongoing disease transmission, using geographic information systems and spatiotemporal statistical analyses, has become indispensable. However, research findings on diarrhea related diseases in Ethiopia have focused solely on prevalence, characteristics of the individuals affected and factors associated with diarrhea [12–16, 25–30]. The findings of these studies are insufficient and fail to capture transmission dynamics in time and space. For effective disease management, knowing when and where disease prevalence reaches a maximum is essential.

The current study investigated spatial, temporal and spatiotemporal patterns of childhood diarrhea in the Amhara region of northwest Ethiopia using spatial SaTScan statistical analysis. Several techniques for spatial analysis had been used previously in different countries to describe geographical patterns and identify clusters of enteric diseases, including diarrhea, in time and space [31–36]. Spatial SaTScan method was used since it is widely suggested that it performs very well in identifying clusters compared to other spatial analysis techniques [37–39], for two reasons. The first is that SaTScan identifies a cluster at any location of any size up to a set maximum, which limits the problem of multiple statistical tests. Second, spatial scan statistics have higher power than other available methods for detecting local clusters [38, 40].

## Materials and Methods

Retrospective data on childhood diarrhea were collected at the district level by reviewing reports made quarterly to the routine surveillance system of the district health office over for a seven year period from July 1, 2007 to June 30, 2014 at the district level.

### Study area

**Fig 1:**
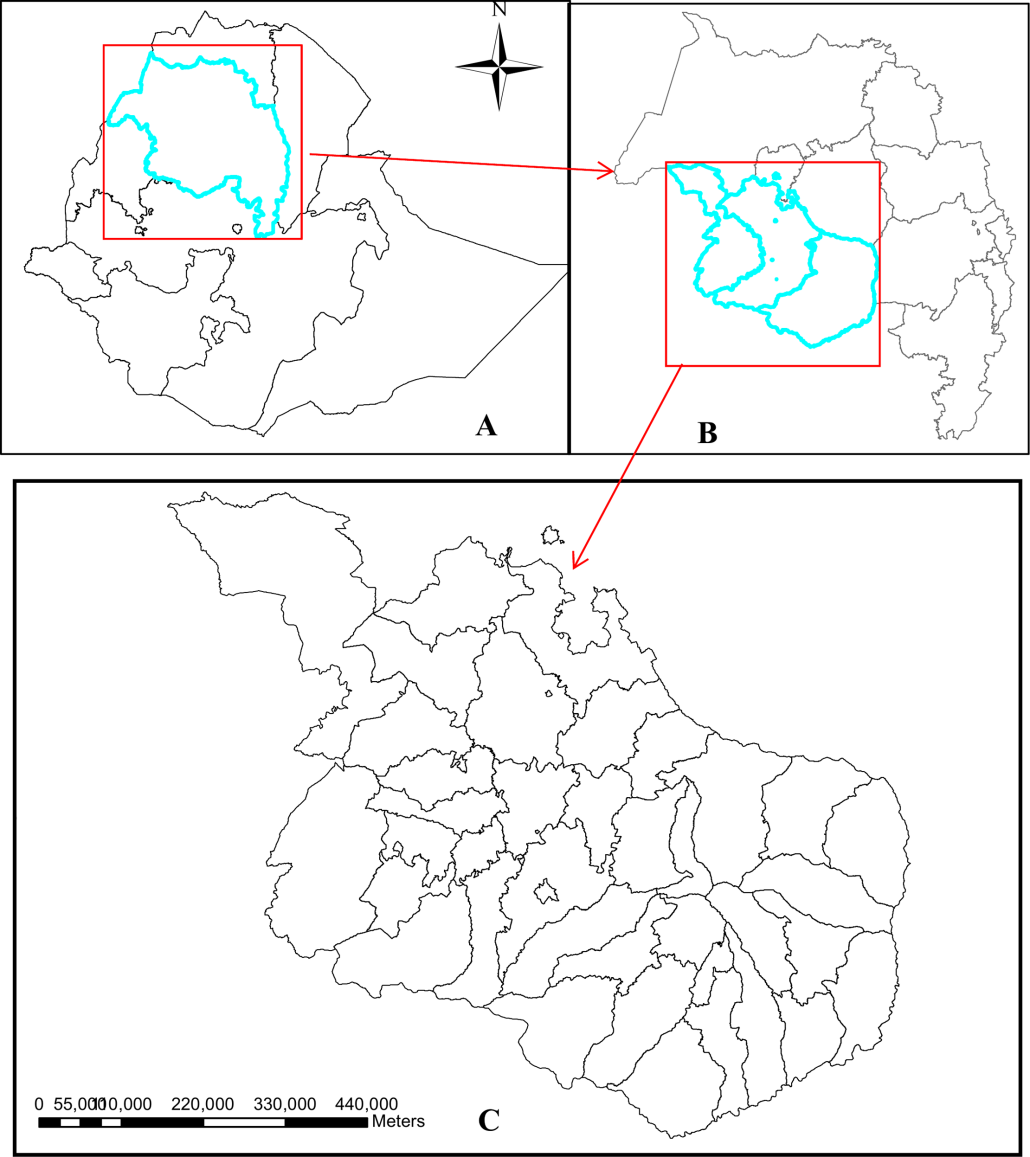
Location of Ethiopia (picture A), Amhara National Regional State (picture B) and the study area (Picture C).

The study area was located in Awi, East Gojjam and West Gojjam administrative zones of the Amhara regional state of Ethiopia, which is in the northwestern part of the country between 9°20' and 14°20' North latitude and 36° 20' and 40° 20' East longitude (Fig 1). Spatiotemporal analysis was conducted in 33 districts in this area. Based on the 2007 National Population and Housing Census of Ethiopia, the projected population for 2013/14 in the study area is about 6,102,870, of which 2,998,149 (49.1%) were male and 826,246 (13.5%) lived in urban areas. Of the total population, 708,430 (11.6%) were children under the age of five years [41]. The study area has two rainy seasons: the main one is from June to September, followed by a shorter one from March to May. The dry season ranges from October to February. The mean maximum and minimum temperatures in the study area are 26°C and 11°C, respectively [42].

### Data collection and data quality control

The number of childhood diarrhea cases was obtained retrospectively from the surveillance system of district health offices over the period of 1 July 2007 to 30 June 2014 (7 years). From July 1, 2007 to June 30, 2013 the data were reported by health institutions to the district health office quarterly (July to September, October to December, January to March and April to June) and from July 1, 2013 to June 30, 2014 they were reported monthly. The health institutions recorded childhood diarrhea cases based on the recommended World Health Organization (WHO) guidelines that define childhood diarrhea as the passage of loose or watery stools at least three times in a 24-hour period [3].

The projected population data of under-five children in a district for each year were obtained from the Amhara Regional State, Bureau of Finance and Economic Development. The population estimate was made based on the results of the 2007 National Population and Housing Census of Ethiopia from the Central Statistics Agency (CSA) [41]. The population density varied across the studied districts over the course of the study period. The projected population data of under-five children were used as the known underlying population at risk to calculate the annual incidence rate of childhood diarrhea. The district level polygon map was obtained from the CSA and spatial data layers were created in ArcGIS 10.0 [43].

A checklist was prepared based on the health management information system registry format to collect the data from the district health management records. To ensure its consistency, the checklist was first prepared in English, translated into Amharic (local language), and then back into English. Health management information system officers were recruited as data collectors. The data collectors were informed about the project’s objectives and trained in data collection procedures. Every day, after collection, the data were checked for completeness and consistency by the principal investigators of the study.

### Data analysis

#### Trend and seasonal distribution analysis

The incidence rate for each quarter for each district was calculated and childhood diarrhea incidences by year and by quarter were plotted to observe seasonal, annual fluctuations and trends in the study area. A smoothing technique was used to reduce data variability that might occur in time series as a result of irregularities and seasonality to observe trends and seasonal distribution of childhood diarrhea. Smoothing was performed based on the moving average window method (using a four-quarter window) [44]. The incidence rate of childhood diarrhea was transformed to log childhood diarrhea rate to satisfy the normality assumption for the linear regression model. Each value (log childhood diarrhea rate) in the chronological series was replaced by a weighted average of this value and neighboring ones to serve as a base for visualizing the trend and the seasonal variation. To estimate the contribution of each season, the weighted average was calculated across all seasons.

#### Spatial distribution of childhood diarrhea

The distribution of excess hazard was described using the standard morbidity ratio, which was calculated as the ratio of the observed cases over the expected cases of a district. The expected cases were derived from the application of the discrete Poisson model on the purely spatial clusters.

#### Spatial, temporal, and space–time cluster analysis

Purely spatial, temporal and spatiotemporal clusters were analyzed using the SaTScan™ spatial statistic developed by Kulldorff [45]. The discrete Poisson model was used as the number of cases of childhood diarrhea in each district was Poisson distributed. Children with diarrhea were taken as cases, and the projected population was the combined number of person-years lived used for fitting the Poisson model [45]. Then, Poisson data were analyzed with the purely spatial, the purely temporal, and the space-time scan statistics.

#### Purely spatial clusters

For detection and analysis of spatial clusters of childhood diarrhea, a purely spatial analysis was conducted using the spatial scan statistic without taking time into account. This spatial statistical analysis method employs the creation of a circular window that scans the entire study area. The radius of the circle varies continuously from zero to a specified maximum size. The maximum-size specified the percentage of the maximum total population at risk within the scanning window. Since researchers recommend the maximum-size be no greater than 50%, that is a reported cluster can contain at most 50% of the total population at risk [40, 46], the maximum cluster size was set to 50% of the population at risk. Thus, numerous overlapping windows of different sizes are generated, which together cover the entire study area. Each circular window is considered a possible candidate cluster. The null hypothesis is that disease risk remains the same inside and outside the scanning window in space, while the alternative hypothesis is that the risk within the window is different from that outside the window. For each circle, the observed cases inside and outside the window are counted and compared to the number of expected cases, as calculated using the Poisson distribution. On this basis, the likelihood ratio within each circle is then calculated. Under the Poisson assumption, the likelihood function for a specific window is proportional to

$$\left({\left(\frac{n}{\mu }\right)}^{n}\right)\left({\left(\frac{N-n}{N-\mu }\right)}^{N-n}\right)\;I\;(n>\mu )$$

Where:

n is the observed number of cases inside the window (district),
μ is the indirectly age-adjusted expected number of cases within the window (in that district) under the null hypothesis,
N is the total number of cases in the study area, and
I is an indicator function that is equal to 1 when the window has more new cases than expected under the null hypothesis and 0 otherwise. It should be noted that n/μ and (N-n)/(N-μ) are proportional to the age-standardized incidence ratios within and outside the window, respectively.

The circle with the maximum likelihood ratio and containing more cases than expected is identified as the most likely (primary) cluster that is least likely to have occurred by chance [45]. The likelihood ratio for this window comprises the maximum likelihood ratio test statistic. The p-value was estimated using Monte Carlo simulations (999) [47], by comparing the rank of the maximum likelihood from the real dataset with the maximum likelihoods from the random datasets [40]. A significance level of alpha < 0.05 was used to test whether the cluster was significant.

#### Spatiotemporal and temporal clusters

The space-time scan statistic was employed to detect clusters in both space and time. This helped us to detect clusters that were not detected by the purely spatial statistic. To detect spatiotemporal clusters a cylindrical window was used. The base of the cylinder represents space, as in the purely spatial scan statistic, while the height reflects time. This statistic behaves as before, except the cylindrical window is then moved in both space and time, so that for each possible geographical location and size, it also visits each possible time interval. For this study, the time window was restricted to annual and the spatial window to 50 kilometers.

As in the previous case, a great many overlapping cylinders of different sizes were thus generated, jointly covering the entire study area and the period studied, with each cylinder reflecting a possible spatiotemporal cluster. Districts with a significant number of cases within the corresponding time were identified using a p-value that was determined using Monte Carlo simulations. Secondary clusters in addition to the most likely cluster (primary) were identified using an iterative manner as detailed in Kulldorff [47] for each purely spatial and space-time scan statistics. In the first iteration, only the most likely cluster was reported in the datasets, then this cluster was removed from the datasets, followed by a second iteration and a completely new analysis using the remaining data. This procedure was then repeated until no clusters remained with p-values of less than 0.05. The maximum cluster size was set to 50% of the population at risk. Purely temporal scan statistics used a window that moved in one dimension only using the height of the cylindrical window as the time dimension. A p-value was generated using Monte Carlo simulations in a manner similar to the spatiotemporal clusters. A significance level of alpha < 0.05 was used to identify a significant district.

Statistical analyses were performed and reported using Excel, SaTScan [48] and ArcGIS 10.0. Excel spreadsheet was used to describe data, draw line graphs and apply smoothing techniques. Spatial and spatiotemporal clusters were analyzed using SaTScan programs. The maps were plotted using ArcGis 10.0.

### Ethical Considerations

Ethical clearance was approved by the research ethics committee of College of Medicine and Health Sciences, Bahir Dar University. A support letter from this institution was given to the Amhara Regional Health Bureau, which in turn issued a support letter to district health offices and requested cooperation for retrieving retrospective data on childhood diarrhea from records. All the information was kept confidential, and no individual identifiers were collected.

## Results

Of the total of thirty six districts in the study area, data were obtained from thirty three districts. Three districts were excluded because of the data incompleteness. Fifteen of the districts were in the East Gojjam administrative zone, eleven in West Gojjam, and the remaining seven in Awi. About 740,764 childhood diarrhea cases were reported from July 1, 2007 to June 30, 2014.

### Distribution and seasonal variation of childhood diarrhea

**Fig 2:**
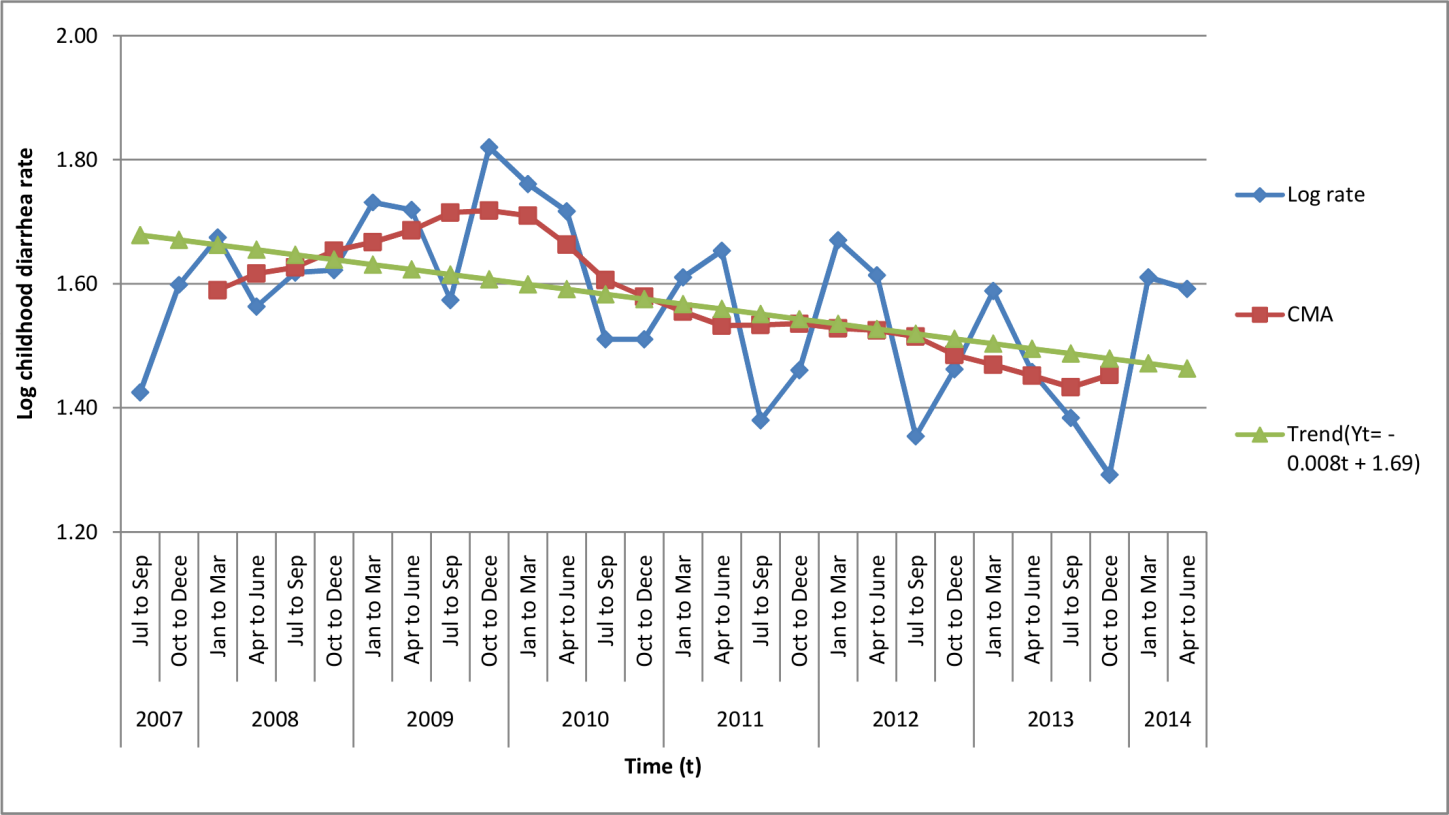
Trend and seasonal variation of childhood diarrhea rate in northwest Ethiopia between 1 July 2007 and 30 June 2014.

Childhood diarrhea during the study period showed a declining trend. A seasonal variation of childhood diarrhea, which peaked from January to March and April to June, was also observed. The result of smoothing using moving average revealed that, in the period between January to March and April to June, 6% and 3% excess childhood diarrhea cases, respectively, were reported from the base of moving average (Fig 2 and S1 File).

The overall average cumulative annual incidence rate during the study period was 155.3 per 1,000 population at risk. The highest incidence (546.8 per 1,000 population at risk) was reported in Dejen from July 1, 2008 to June 30, 2009, while the lowest occurred in Wenberma district (40.5 per 1,000 population at risk) from July 1, 2012 to June 30, 2013. The distribution pattern of childhood diarrhea varied greatly across the study districts. Most had higher incidence rates, 100 or more cases per 1,000 population at risk (Table 1).

**Table 1:** Annual childhood diarrhea incidence rate at the district level in northwest Ethiopia between 1 July 2007 and 30 June 2014.

Name of district	2007/8	2008/9	2009/10	2010/11	2011/12	2012/13	2013/14
Bibugn	115.9	108.4	131.7	82.5	82.2	71.1	73.3
Senan	66.6	151.1	346.7	282.7	158.4	97.9	131.1
Mechkel	202.4	265.2	251.9	394.7	275.3	194.9	239.3
Debere Elias	282.9	328.8	374.4	189.6	122.4	162.8	151.0
Gozamin	210.9	325.4	327.3	431	262.1	154.0	182.4
Baso Liben	117.3	188.9	216.1	117.9	111.5	96.1	71.7
Aneded	63.2	121.0	198.2	328.1	191.1	114.3	106.3
Awabel	161.7	160.3	366.6	85.8	98.0	66.2	70.5
Dejen	223.7	546.8	444.6	199.6	179.2	127.4	126.4
Enemay	164.0	136.0	432.4	189.3	252.7	137.5	138.4
Shebel Berenta	86.9	159.3	160.8	84.2	75.7	90.4	99.3
Enarj Enawuga	196.7	162.1	289.6	183.6	158.6	146.4	127.2
Gonchasiso Enese	252.0	426.0	209.7	108.2	92.7	69.2	49.9
Enebise SarMidir	195.7	317.3	212.8	162.0	199.3	184.6	183.4
Huletej Enese	145.0	181.1	289.8	351.6	251.5	76.5	114.8
Bahir Dar Zuriya	63.4	61.6	76.1	90.3	69.2	43.6	74.1
Bure	256.5	194.4	164.3	141.1	148.7	94.2	134.7
South Achefer	116.5	138.9	222.4	148.7	110.3	181.2	212.8
Dega Damot	119.0	89.9	73.0	65.2	75.7	133.0	82.4
Dembecha	120.0	189.8	243.0	148.4	157.9	106.9	133.3
Jebitenan	305.6	289.1	152.6	127.5	191.8	188.7	180.2
Mecha	161.1	138.4	173.1	128.8	111.6	162.2	137.3
Quarit	90.0	289.0	99.2	85.7	207.4	207.5	76.3
Sekela	79.7	87.0	119.4	64.9	98.8	61.8	76.3
North Achefer	100.2	109.6	102.9	88.6	126.5	67.4	79.2
Wenberma	126.2	104.4	104.3	102.3	146.4	40.5	111.6
Dangila	102.1	190.4	220.0	56.3	70.2	73.7	106.8
Fagita Lekoma	82.9	73.2	82.7	53.0	86.9	87.7	95.4
Guagusa Shekudad	122.4	123.8	286.5	52.4	146.3	132.3	79.8
Guangua	159.2	199.3	175.0	108.1	104.6	148.9	205.3
Jawi	130.8	128.0	327.6	85.0	98.7	137.8	218.9
Banja Shekudad	189.4	116.5	113.4	104.5	109.5	172.6	138.7
Anikasha Gwagusa	148.8	159.1	54.5	122.4	72.5	101.8	72.2

### Spatial distribution of childhood diarrhea

**Fig 3:**
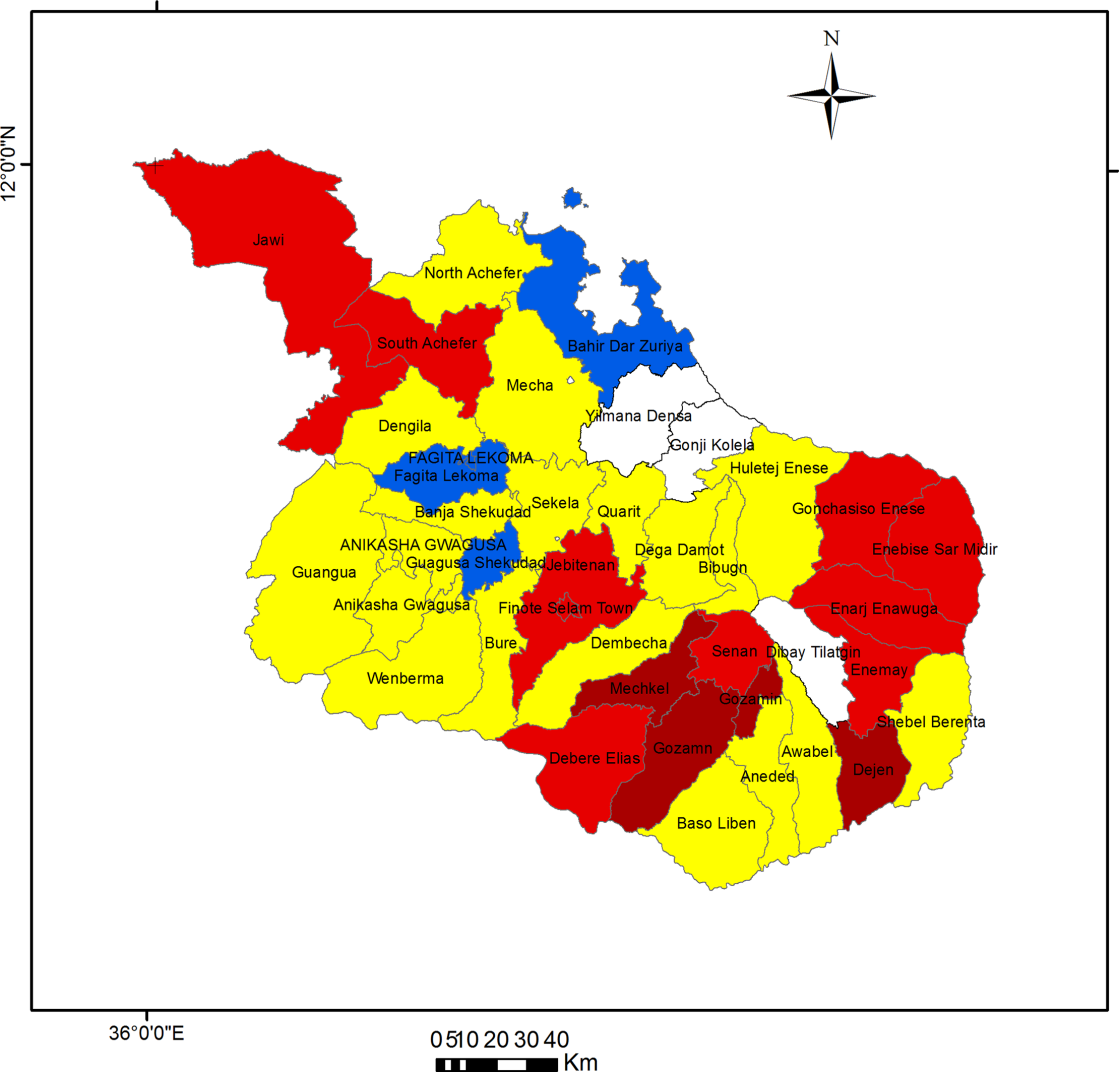
Excess risk map of childhood diarrhea in northwest Ethiopia, 1 July 2007 to 30 June 2014. Dark red color indicates districts with standard morbidity ratio (SMR) between 1.51 and 2.00, light red indicates SMR between 1.01 and 1.50, yellow color indicate SMR between 0.51 and 1.00, and blue color indicate SMR < = 0.50.

The excess hazard map shows the distribution of excess risk, which was defined as the ratio of the number of observed over the number of expected cases. Areas in blue (2 districts) and yellow (14 districts) had a lower risk than expected, as indicated by the excess risk of values of <1. In contrast, districts colored dark red (3) and red (14) had higher than expected or excess risk (>1). Of the 17 districts with excess risk, 11 were located in the East Gojjam administrative zone, 2 in Awi zone, and 4 were in West Gojjam (Fig 3 and S2 File).

### Purely spatial clusters of childhood diarrhea

**Fig 4:**
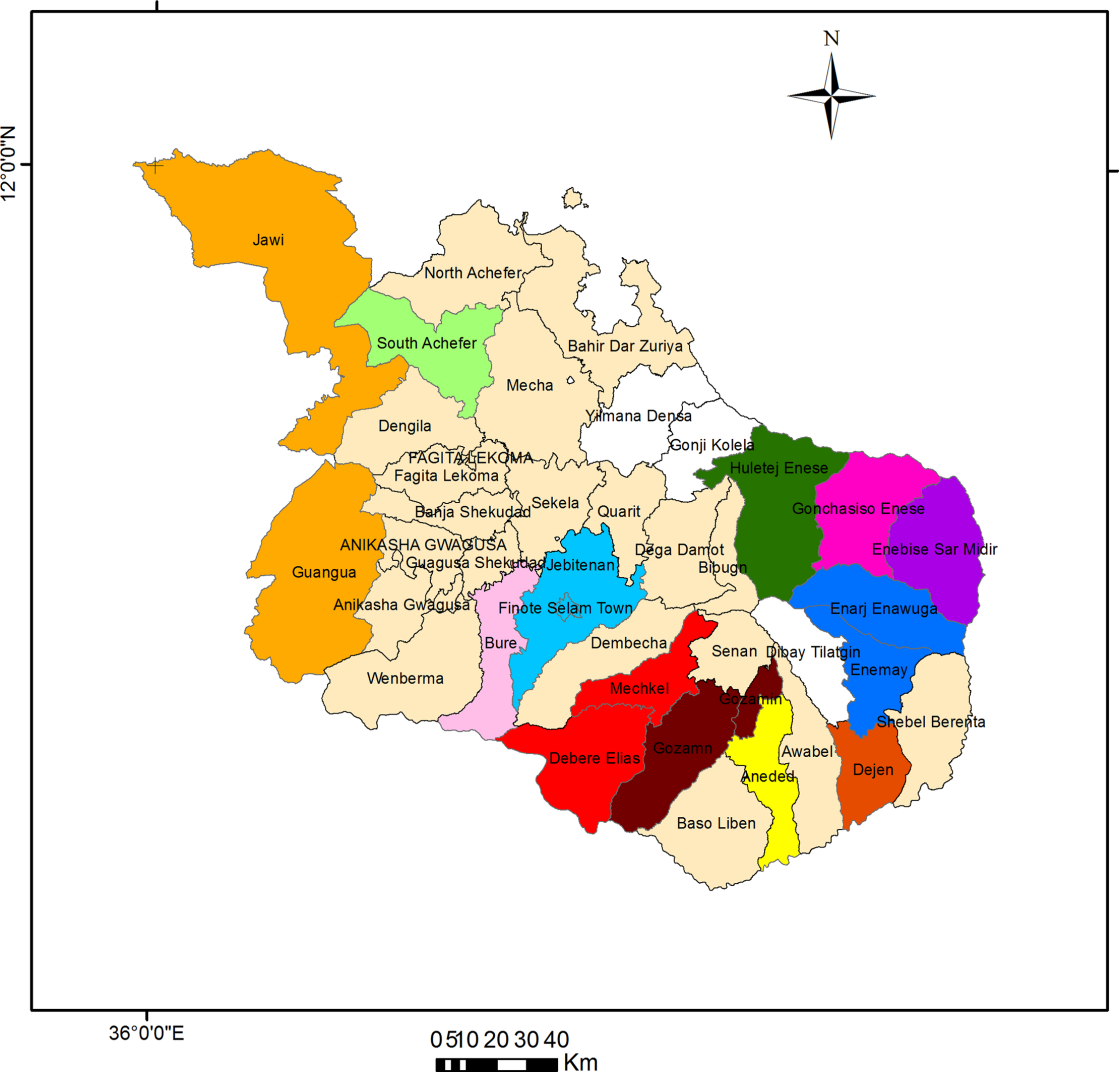
Most likely clusters (1) and secondary cluster (2, 3, 4, 5, 6, 7, 8, 9, 10, 11 and 12) of childhood diarrhea in northwest Ethiopia between 1 July 2007 and 30 June 2014. Numerical identification of the clusters are in order of their likelihood ratio; Dark red color indicates the cluster with the highest likelihood ratio and labeled cluster 1 (most likely cluster or primary cluster) while cluster 2 (light red color), cluster 3 (flame red color) and cluster 4 (green), cluster 5 (light blue), cluster 6 (dark blue), cluster 7 (violet), cluster 8 (pink), cluster 9 (light apple), cluster 10 (gold), cluster 11 (rose) and cluster 12 (yellow) are secondary clusters from the highest to lowest likelihood ratio. Beige color indicates none cluster districs. For more detailed cluster information, refer to Table 2.

The spatial pattern of childhood diarrhea was found to be nonrandom. A total of 12 clusters (1 most likely cluster and 11 secondary clusters) were identified throughout the study period, 8 of which were located in East Gojjam, and 3 in West Gojjam. The remaining 1 cluster was in the Awi administrative zone. The most likely cluster was observed in the East Gojjam zone of Gozamin district. The cluster window was centered at 10.349433 N, 37.729800 E. The base diameter of the window was 0 km, with a Relative Risk (RR) of 1.85. The Log Likelihood Ratio (LLR) for the most likely cluster was 7123.89, p <0.001. Secondary clusters were located in Aneded, Debere Elias/Mechkel, Dejen, Enarj Enawuga/Enemay, Enebise SarMidir, Gonchasiso Enese, Huletej Enese, and, all of which are found in the East Gojjam zone; Bure, Jebitenan, and South Achefer in the West Gojjam zone; and Gunagua/Jawi in the Awi zone (Table 2 and Fig 4).

**Table 2:** Spatial clusters of childhood diarrhea in northwest Ethiopia between 1 July 2007 and 30 June 2014.

Cluster	District	Population	Coordinates / radius	Obs.^*^	Exp.^†^	RR	LLR	p-value
1	Gozamin	178250	10.349433 N, 37.7298 E/0 km	47933	26711.53	1.85	7123.89	<0.001
2	Debere Elias	93038	10.292258 N, 37.457120 E/19.59 km	51599	31426.64	1.69	5702.40	<0.001
2	Mechkel	116679	10.437225 N, 37.55905 E/19.59 km	51599	31426.64	1.69	5702.40	<0.001
3	Dejen	74995	10.165850 N, 38.150583 E /0 km	19570	11238.15	1.76	2571.06	<0.001
4	Huletej Enese	262455	11.08010 N, 37.677380 E /0 km	52649	39329.63	1.36	2163.98	<0.001
5	Jebitenan	206270	10.674700 N, 37.255545 E/0 km	42010	30910.27	1.38	1876.98	<0.001
6	Enarj Enawuga	170906	10.65818 N, 38.1658 E/23.75 km	53413	42066.58	1.29	1501.26	<0.001
6	Enemay	109814	10.44735 N, 38.20133 E/23.75 km	53413	42066.58	1.29	1501.26	<0.001
7	Enebise SarMidir	138476	10.871260N, 38.270080 E /0 km	28681	20751.01	1.40	1396.15	<0.001
8	Gonchasiso Enese	159997	10.90333 N, 38.086 E/0 km	27081	23975.94	1.13	199.64	<0.001
9	South Achefere	160610	11.361433 N, 36.963633 E / 0 km	26045	24067.8	1.09	81.8	<0.001
10	Guangua	166038	10.953633 N, 36.502445 E/ 39.7km	40882	38640.1	1.06	67.3	<0.001
10	Jawi	91815	10.621066 N, 36.369138 E/39.7 km	40882	38640.1	1.06	67.3	<0.001
11	Bure	144671	10.704768 N, 37.058193 E/ 0 km	23214	21679.4	1.07	54.7	<0.001
12	Aneded	103125	10.252640 N, 37.843733 E/0 km	16555	15453.51	1.07	39.19	<0.001

RR, Relative risk; LLR, Log likelihood ratio

* Number of observed cases in a cluster

† Number of expected cases in a cluster.

### Spatiotemporal clusters of childhood diarrhea

The most likely spatiotemporal cluster was located in all districts of the East Gojjam administrative zone (Aneded, Awable, Basoliben, Bibugn, Debre Elias, Dejen, Enarj Enawuga, Enebise SarMidir, Enemay, Gonchasiso Enese, Gozamin, Huletej Enese, Mechkel, Senan, and Shebel Berenta,) and three districts of the West Gojjam zone (Dega Damot, Dembecha, and Jebitenan) (LLR = 24929.90, p<0.001) from July 1, 2009 to June 30, 2011. Secondary clusters were identified in Jawi (LLR = 1015.63, p<0.001), and Dangila/South Achefer (LLR = 685.18, p<0.001), from July 1, 2009 to June 30, 2010 and Quarit (LLR = 815.70, p<0.001), from July 1, 2008 to June 30, 2009 (Table 3).

**Table 3:** Spatiotemporal clusters of childhood diarrhea in northwest Ethiopia between 1 July 2007 and 30 June 2014.

	District	Time frame	Observed^*^	Expected^†^	RR	LLR	p-value
Most likely cluster	Gozamin/Senan/Mechkel/Aneded/Awable/Basoliben/Dejen/Debre Elias/Dega Damot/Huletej Enese/Bibugn/Jebitenan/Enemay/Dembecha/Enarj Enawuga/Shebel Berenta/Gonchasiso Enese/Enebise SarMidir	July 1 2009 to June 30 2011	236660	154315.24	1.78	24929.90	<0.001
Secondary cluster	Jawi	July 1 2009 to June 30 2010	4219	1928.74	2.19	1015.63	<0.001
Secondary cluster	Quarit	July 1 2008 to June 30 2009	4627	2397.86	1.94	815.70	<0.001
Secondary cluster	Dangila/South Achefer	July 1 2009 to June 30 2010	10111	6845.94	1.48	685.18	<0.001

RR, Relative risk; LLR, Log likelihood ratio

^*^ Number of observed cases in a cluster

^†^ Number of expected cases in a cluster.

### Purely temporal clusters of childhood diarrhea

Significantly higher rate of purely temporal childhood diarrhea cluster was detected. Only one peak period of an identified primary cluster occurred in all districts (LLR = 9655.86, p = 0.001) during July 1, 2008 to June 30, 2010.

## Discussion

The trend of childhood diarrhea showed a declining pattern in space and time in northwest Ethiopia during the study period. This might be due to Water, Sanitation and Hygiene (WASH) interventions carried out by the government and non-governmental organizations [49, 50]. Interventions with WASH seem well progressing in Ethiopia in all these areas in the past decade. The majority of the population, however, still did not have access to improved water supply (51%) [9] and did not use improved sanitation (79%) in 2011 [51]. In spite of the slight decline, the annual incidence rate of childhood diarrhea relatively remained to be high in most districts. Such a high rate may be attributable to many factors in addition to the use of unimproved water supply and unimproved sanitation, such as poor hand hygiene, poor food hygiene, mothers’ limited education, improper house hygiene, and malnutrition [13, 22, 52–55].

A trend of seasonality in peak childhood diarrhea cases, which occurred more frequently from January to March and April to June, was also observed. In the study area, the period between January and March is a dry season and April to June is the beginning of the rainy season, both of which exhibit high temperatures compared to other months [56]. This finding is consistent with previous studies [21, 57–62] that showed climate factors significantly affected seasonal childhood diarrhea, with more cases observed during the periods of high temperature and drier months of the year, for a number of reasons. Increased ambient temperature favors the growth of bacterial and parasitic diarrhea and extends the survival of enterogastritis-causing bacteria in the external environment [63–66]. Environmental conditions such as surface run off and flooding increase the rate of contamination of drinking water sources at the beginning of the rainy season, as the first rainfall events after the dry season wash contaminants from the surface into the water sources [67]. The practice of open defecation is a common practice in Ethiopia, including the study area [51]. A growing concern of accessing to unsafe drinking water was found that 60% of springs and 45% of well water had excessive E.coli in a national study [68]. The dry season indirectly creates temporal changes in human behavior, such as increased consumption of water because of higher ambient temperatures. Drinking water scarcity may lead to a decline in hygiene practices, which in turn promotes the transmission of diarrhea [57, 67]. During the dry season, most hand dug wells and springs are out of commission, which forces households to use unimproved water sources such as rivers and ponds [67].

Another important finding of this study was that the spatial, temporal, and space-time distribution patterns of childhood diarrhea cases in the study districts were nonrandom. The purely spatial model identified one most likely cluster in the East Gojjam Zone. The spatiotemporal models indicated that the most likely cluster was located in all districts of East Gojjam zone and in three districts of the West Gojjam zone that neighbor East Gojjam. Moreover, the East Gojjam zone also contained seven secondary clusters identified by the spatial model and two secondary clusters in neighboring West Gojjam. In short, the study identified clusters that are closely related to a specific geographical area in the same region and share similar geographical parameters, such as altitude and weather conditions, and economic characteristics. Since the importance of cluster analysis in epidemiology is to detect aggregation of disease cases and ultimately to find evidence of risk factors on which prevention and control activities can be focused, the area in which the identified clusters were found should be given priority for childhood prevention and control interventions [69].

The spatiotemporal analysis provided further evidence for a higher than expected number of childhood diarrhea cases arising within a defined place and time period. The most likely spatiotemporal cluster was found in all districts of the East Gojjam zone, and in the Dega Damot, Dembecha, and Jebitenan districts of the West Gojjam zone between July 1, 2009 and June 30, 2011. Secondary space-time clusters were also identified between July 1, 2009 and June 30, 2010 in Dangila, Jawi, and South Achefer districts and in Quarit district on July 1, 2008 to June 30, 2009. The occurrence of clusters before 2012 may be explained in part by the commencement in 2011 of water, sanitation and hygiene interventions with community mobilization by the government [70].

Finally, this study detected significantly higher rates of purely temporal childhood diarrhea cluster from July 1, 2008 to June 30, 2010, in all districts. The reduction of childhood diarrhea following this occurrence of high incidence could also be due to the government interventions addressing drinking water supply and sanitation and the impact of health extension interventions using different approaches to behavioral changes and communication. For instance, recent evidence showed the provision of improved water supply had been increased from 29% in 2000 to 54% in 2011 [9, 10]; the use of improved sanitation had increased from 8% in 2000 to 22% in 2011 [51]; and the prevalence of open defecation had decreased from 61% in 2005 to 39% in 2010 [71]. Recent studies showed significantly greater decreases in childhood diarrhea in model households than in non-model households following intervention with health extension packages [12, 14]. One strategy to implement such packages is to provide every household with a minimum of 72 hours of training to bring positive behavioral change on hygiene and sanitation. This will be an important step toward the maintenance of a healthy environment through the raising of house to house health awareness with active community participation.

A limitation of this study is the incompleteness and non-representativeness of the data on childhood diarrhea. This limitation is believed to be minimal since the drawback of secondary data across all districts is more likely the same. Due to unavailability of data, this study could not test the association between incidence of childhood diarrhea and temperature, rainfall, improved water supply or sanitation. The spatial scan statistics method is highly efficient in detecting local clusters with good accuracy and can help epidemiologists or public health practitioners to evaluate disease clusters and early detection of disease outbreaks. This technique has been used to detect, analyze, and characterize the spatial, temporal, and spatiotemporal patterns of diarrhea clusters in recent years [69, 72–75]. Clusters in this method are assumed to be circular and dissimilar in size to areas within given district boundaries. This could result in the exclusion or inclusion of districts that register excess or less risks. The cluster analysis can, however, provide valuable information about spatial disparity of childhood diarrhea that may be relevant for further study in the study area. The results of this study will allow researchers to generate a hypothesis as to the contextual environmental or socioeconomic factors that might be influencing the distribution of childhood diarrhea. The identification and understanding of clusters in space and time at the village and agro-ecology are also areas for future research.

## Conclusions

This study showed statistical evidence of spatial, temporal and spatiotemporal clustering, as well as seasonal patterns and decreasing temporal trends, in the distribution of childhood diarrhea cases in the study area. The advanced spatial statistical methods used constitute complementary tools for epidemiological surveillance of infectious diseases to guide prevention and control efforts. Thus, analyzing surveillance data using such techniques can provide useful information for public health planners in decision making by identifying underserved populations and allocating scarce resources for appropriate locations to implement specific interventions such as provision of safe drinking water supply, promotion of proper environmental and house hygienic practices, proper food hygienic and water handling practices. Taking into account the spatiotemporal variation in the study areas is highly recommended when striving to design an effective and efficient childhood diarrheal disease intervention strategies.

## Supporting Information

S1 File Smoothing using moving averages to visualize the trend of childhood diarrhea. (XLS)

S2 File Observed, expected cases and excess risk in each district. (XLS)

## Abbreviations

CSA: Central Statistics Agency
LLA: Log Likelihood Ratio
RR: Relative risk
SSA: Sub-Saharan African
WHO: World Health Organization
WASH: Water, Sanitation and Hygiene
